# Supplementary material for: In silico analysis of local RNA secondary structure in influenza virus A, B and C finds evidence of widespread ordered stability but little evidence of significant covariation
Source: Sci Rep. 2022 Jan 10;12:310. doi: 10.1038/s41598-021-03767-x (PMC8748542; doi:10.1038/s41598-021-03767-x)
Supplement: Supplementary file 1 — Supplementary Information. [file 41598_2021_3767_MOESM1_ESM.zip › Supplemental Files/Supplemental File 3.zip!/Supplemental File 3/CM-AAll/pdf/IAV_seg4_1_1.fold.R2R.sto.pdf]

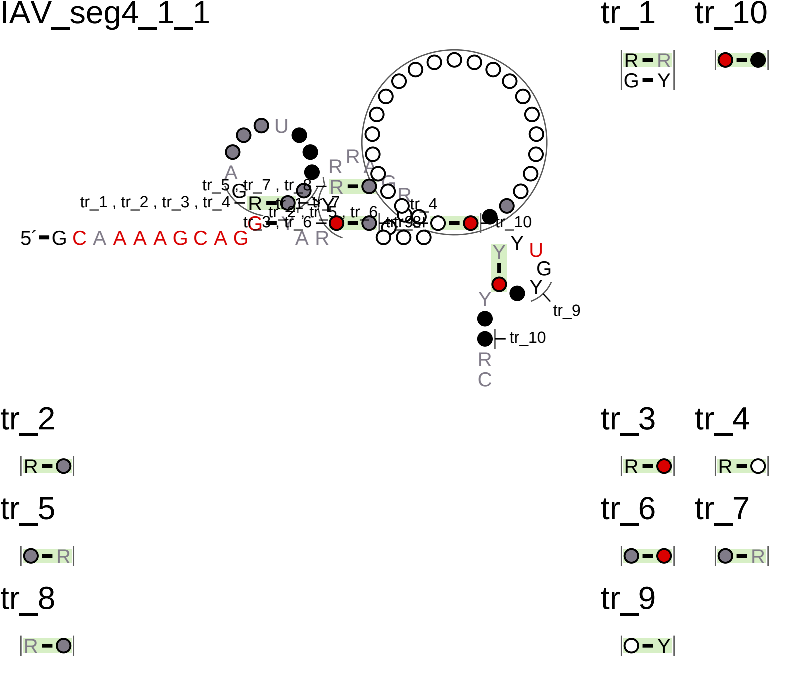

IAV_seg4_1_1
tr_1
tr_10
R R
G Y
tr_5 , tr_7 , tr_8
tr_1 , tr_2 , tr_3 , tr_4
5´ G C A A A A G C A G
A R
tr_4
tr_10
Y U
G
Y
tr_9
Y
tr_10
R
C
tr_2
R
tr_3
R
tr_4
R
tr_5
R
tr_6
tr_7
R
tr_8
R
tr_9
Y
